# Accurate Structural Correlations from Maximum Likelihood Superpositions

**DOI:** 10.1371/journal.pcbi.0040043

**Published:** 2008-02-15

**Authors:** Douglas L Theobald, Deborah S Wuttke

**Affiliations:** 1 Department of Biochemistry, Brandeis University, Waltham, Massachusetts, United States of America; 2 Department of Chemistry and Biochemistry, University of Colorado at Boulder, Boulder, Colorado, United States of America; Fox Chase Cancer Center, United States of America

## Abstract

The cores of globular proteins are densely packed, resulting in complicated networks of structural interactions. These interactions in turn give rise to dynamic structural correlations over a wide range of time scales. Accurate analysis of these complex correlations is crucial for understanding biomolecular mechanisms and for relating structure to function. Here we report a highly accurate technique for inferring the major modes of structural correlation in macromolecules using likelihood-based statistical analysis of sets of structures. This method is generally applicable to any ensemble of related molecules, including families of nuclear magnetic resonance (NMR) models, different crystal forms of a protein, and structural alignments of homologous proteins, as well as molecular dynamics trajectories. Dominant modes of structural correlation are determined using principal components analysis (PCA) of the maximum likelihood estimate of the correlation matrix. The correlations we identify are inherently independent of the statistical uncertainty and dynamic heterogeneity associated with the structural coordinates. We additionally present an easily interpretable method (“PCA plots”) for displaying these positional correlations by color-coding them onto a macromolecular structure. Maximum likelihood PCA of structural superpositions, and the structural PCA plots that illustrate the results, will facilitate the accurate determination of dynamic structural correlations analyzed in diverse fields of structural biology.

## Author Summary

Biological macromolecules comprise extensive networks of interconnected atoms. These complex coupled networks result in correlated structural dynamics, where atoms and residues move and evolve together as concerted conformational changes. The availability of a wealth of macromolecular structures necessitates the use of robust strategies for analyzing the correlated modes of motion found in molecular ensembles. Current strategies use a combination of least-squares superpositions and statistical analysis of the structural covariance matrix. However, the least-squares treatment implicitly requires that atoms are uncorrelated and that each atom has the same positional uncertainty, two assumptions which are violated in structural ensembles. For example, the atoms in the proteins are connected by chemical bonds, covalent and non-covalent, resulting in strong correlations. Furthermore, different atoms have different variances, because some atoms are known with less precision or have greater mobility. Using maximum likelihood (ML) analysis, we have developed a technique that is markedly more accurate than the classical least-squares approach by accounting for both correlations and heterogeneous variances. The improved ability to accurately analyze the major modes of dynamic structural correlations will benefit a diverse range of biological disciplines, including nuclear magnetic resonance (NMR) spectroscopy, crystallography, molecular dynamics, and molecular evolution.

## Introduction

Biological macromolecules, like proteins and catalytic RNAs, are dynamic structures. Each of the atoms in a macromolecule is coupled with other atoms via covalent bonds and various non-covalent interactions. This large and complex network of interconnections produces correlated structural dynamics, in which a perturbation or movement of one structural element covaries with the positional displacement of other elements. Thus, over a given time frame, macromolecules exist as an ensemble of correlated substates which span a large configurational space. Relevant time scales for dynamic structural change can range from picoseconds in molecular dynamics studies, to milliseconds for large structural movements, to millennia in evolutionary analyses of conformational perturbations due to amino acid substitutions. Understanding the correlated dynamics of such systems is essential for mapping structure to function. However, structural biologists currently have few tools for analyzing the correlations found in an ensemble of structures.

Previous work characterizing the structural correlations in macromolecules has been limited to analysis of molecular dynamics (MD) simulations. Two general methods have been used to extract major modes of functionally relevant motions: normal mode analysis [1] and principal components analysis (PCA) of atomic covariance matrices [2,3]. Studies using these methods have largely shown that protein motions are dominated by only a few major distinct modes of correlated movement. Normal mode analysis assumes that dynamics are harmonic. In contrast, PCA does not make this assumption, and it has been found to be useful for finding major modes when the dynamics are highly anharmonic, which is more biologically realistic since proteins have multiple energetic minima [1].

In standard practice, PCA of an MD trajectory first involves removal of arbitrary rotational and translational effects by conventional least-squares superpositioning [4–8]. From this least-squares superposition one then calculates a covariance matrix, which is subsequently used as input for eigendecomposition in PCA (also see [9]). However, the use of least squares is problematic in both theory and practice. As a statistical technique, least squares relies on two strong physical assumptions: that all atoms have the same variability, and that each atom is uncorrelated with the others. When these assumptions do not hold, least squares can give very misleading results [10]. In biomolecular applications, individual atoms in a superposition do not have equal variances, as some regions superposition closely while others show more conformational heterogeneity. Similarly, the atoms in macromolecular structures are strongly correlated by physical coupling via chemical bonds. Thus, both of the assumptions of least squares are violated in real biological data. In fact, performing PCA of a least-squares superposition is logically contradictory; the least-squares method assumes that no correlations exist, yet PCA is then performed on the least-squares derived covariance matrix to analyze those “nonexistent” correlations.

We use a maximum likelihood (ML) method that overcomes the drawbacks of conventional least-squares superpositioning methods [11–13]. Unlike least squares, ML superpositioning is valid in the presence of heterogeneous variances and correlations, thereby providing more accurate superpositions [12,13] and corresponding covariance (and correlation) matrices. Rather than performing separate superpositioning and covariance matrix calculation steps, our ML superpositioning method simultaneously determines the optimal superposition and the optimal covariance matrix. We show that, as expected, PCA of our ML superposition provides markedly more accurate structural correlations than those extracted from least-squares superpositions. Furthermore, we show that use of the correlation matrix, rather than the covariance matrix, automatically corrects for biases that may be introduced due to experimental uncertainty in atomic positions or due to large differences in the magnitude of dynamic motion. We provide examples of the generality of the method by applying it to alternate crystal forms of the same protein, nuclear magnetic resonance (NMR) ensembles, and distant homologs with differing amino acid sequences.

## Results/Discussion

### Accuracy of ML Correlation Matrices

We performed two simulation analyses to confirm the ability of our ML method to accurately determine the structural correlations found in sets of conformationally similar molecules. Two sets of conformationally perturbed protein structures were generated randomly by assuming a Gaussian distribution with known mean and known covariance matrices (and, hence, based on known correlation matrices; see Figure 1A and 1E). In this case, the covariance matrix is a mathematical description of the positional variation and correlations among the atoms in an ensemble of molecular structures (for more background regarding covariance and correlation matrices, see Methods). Two different covariance matrices were used: one with a range of variances, yet no correlations, and another with the same range of variances plus strong correlations (the corresponding “true” correlation matrices are plotted in Figure 1A and 1E). The correlation structure and the range of variances are typical of NMR solution structures found in the PDB database (see Methods). We then randomly translated and rotated each of the perturbed structures. Both least-squares and ML superpositions were performed independently on these two sets of simulated protein structures to obtain estimates of the true covariance/correlation matrix that was used to generate the structures (Figure 1B–1D and 1F–1H).

**Figure 1:**
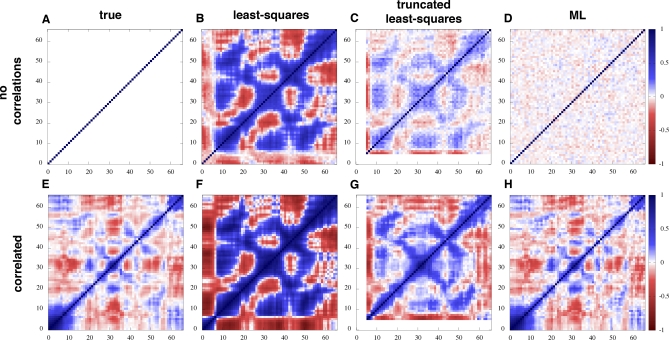
Plots of Correlation Matrices Inferred from Superpositions The upper row (A–D) shows plots in which the true correlation matrix has no correlations (all off-diagonal elements are exactly zero), whereas the lower row (E–H) shows plots where the true correlation matrix has strong, complex, positive, and negative correlations. Positive correlation, zero correlation, and negative correlation are represented by colors ranging from blue to white to red, respectively. (A, E) The true, assumed correlation matrix used in the simulation. (B, F) The correlation matrix calculated from a least-squares superposition, including all atoms, of 300 protein structures simulated using the true correlation matrix. (C, G) The estimated correlation matrix, calculated from a least-squares superposition that omitted residues 1–5, which have the highest variance (most disorder) in the structure. (D, H) The correlation matrix calculated from a maximum likelihood superposition of the same simulated structures.

We found that, when calculated from an ML superposition, both the covariance matrix and the corresponding correlation matrix are considerably more accurate than those calculated from least-squares superpositions (Figure 1). When compared to the true (known) correlation matrix, the least-squares correlation matrix is highly biased, showing an artifactual pattern of correlation (Figure 1B and 1F). As shown in Figure 1C and 1G, the least-squares correlation matrix remains artifactually biased even when the majority of highly variable atoms are excluded from the analysis, as often done in common practice (“truncated least squares,” where disordered regions are subjectively removed from the analysis with intent to obtain lower RMSDs). Interestingly, the least-squares procedure imparts a highly similar, artifactual correlation structure regardless of the true correlations (compare Figure 1B and 1C, with no true correlations, to Figure 1F and 1G, in which the structures had true strong correlations). In contrast, the ML-based correlation matrix reliably recapitulates the true complex patterns of correlation (Figure 1D and 1H).

### Accuracy of Major Modes of Correlation Found by ML

To extract major modes of structural correlation from a superposition, we use the statistical method of principal components analysis (PCA; see Methods). PCA produces multiple principal components, each of which represents the predominant modes of structural correlation within the superposition. Generally, only the first few principal components (that is, those with the largest eigenvalues) are of practical interest, since they usually account for the majority of correlations in the data. As shown below, when significant covariation exists in a family of structures, PCA based on a least-squares superposition will yield erroneous principal components, resulting in artifactual modes of correlation.

As with the correlation matrices, we found that the principal components determined from an ML superposition are likewise more accurate than principal components from a least-squares superposition (Figure 2). In these images, the largest (or first) principal component has been plotted in color on a single representative structure from the superposition. We refer to these types of graphs as “PCA plots.” Red regions are correlated with each other, meaning that these regions tend to “move together” on average within the set of structures. Similarly, blue regions are also correlated with each other. However, the red regions are anti-correlated with the blue regions, meaning that red and blue regions tend to “move” in opposition to each other. White regions represent atoms whose positions are completely uncorrelated.

**Figure 2:**
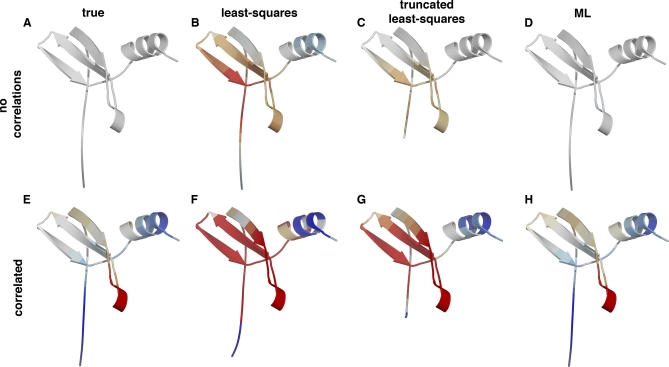
PCA Plots of Least-Squares and ML Superpositions of Simulated Structures The first principal component (PC) is plotted on the mean structure for various calculations. As in Figure 1, the upper row (A–D) uses a covariance matrix in which all correlations are zero (no correlation), whereas the lower row (E–H) uses a covariance matrix with strong correlations. Red regions are self-correlated, as are blue regions, while blue versus red regions are anti-correlated. White regions indicate no correlation. (A, E) The true first PC, extracted from the known correlation matrices. (B, F) The first PC based on the all-atom least-squares superposition. (C, G) The first PC from a least-squares superposition that excluded residues 1–5 with the largest variance in the simulation. (D, H) The first PC based on the ML superposition.

In the first analysis, the PCA plots shown in Figure 2A–2D were calculated from simulated structures that had no bona fide correlations among their atoms (using the correlation structure plotted in Figure 1A). Nevertheless, the largest principal components from the least-squares superpositions indicate a substantial, yet completely artifactual, mode of correlation, even when only the well-ordered residues are included in the superposition (compare the true first principal component in Figure 2A with Figure 2B and 2C). In contrast, the first principal component from the ML superposition faithfully shows very little correlation, as indicated by the lack of colored patterns (Figure 2D). PCA of the ML superposition also avoids the need for a subjective judgment on which residues to remove from the analysis.

In the second, complementary analysis, protein structures were simulated which had strong correlations, using the correlation matrix plotted in Figure 1E. As before, the first principal component from the least-squares superposition indicates a large, artifactual mode of correlation, which is still present even when the highly variable residues are excluded (Figure 2F and 2G). PCA of the ML superposition, however, accurately estimates the true correlation (Figure 2H).

Results from our ML method differ most from the conventional least-squares method when there is a wide range of variances among the atoms (that is, when some regions of the structures are well-superpositioned and other regions are highly disordered) and when correlations are strong. As the variances for the atoms become more uniform, and as the correlations approach zero, our method converges on the conventional least-squares method. Even so, the poor performance of the least-squares PCA method persists despite the removal of the majority of the most highly variable residues (residues 1–5 at the N-terminus; see Figure 2C and 2G). Thus, with the improved accuracy of ML superpositions, PCA can be used reliably to find the major modes of positional variation and dynamical correlation within a family of structures.

### Structural Correlations from Alternate Crystal Structures: The 70S Ribosomal Subunit

The method presented here for identifying major modes of structural correlation is general, and in principle it can be used to analyze any structural superposition, including independent solutions of the same protein, different homologous proteins, or a series of MD conformations. As one example, Figure 3 shows the second principal component from an ML superposition of a series of 10 crystal structures structures of the 70S ribosomal subunit from Haloarcula marismortui, including nine structures of the subunit bound to different antibiotics [14–16]. Remarkably, the majority of the correlation is localized to the active site of ribosome, the subunit interface, and the active site cleft, which binds the actively transcribed mRNA, tRNAs, translation factors, and the nascent polypeptide. The regions of strong correlated positional displacement also roughly correspond to regions of high RNA sequence conservation (see, for example, Figure 5 of [14]). Thus, this PCA plot suggests that conformational perturbations of the ribosome during binding by various antibiotics are accompanied by correlated changes in distant yet functionally important regions.

**Figure 3:**
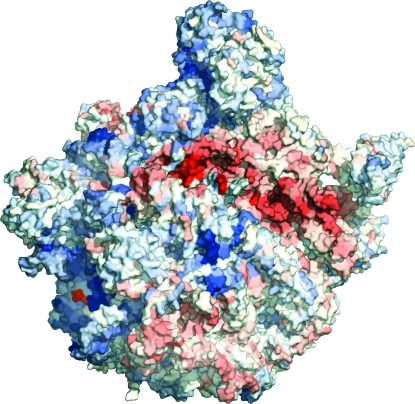
PCA Plot of the 70S Ribosomal Subunit from Haloarcula marismortui The second principal component is plotted for a superposition of 10 subunits of the large ribosomal subunit bound to different antibiotics. The subunit interface (which binds the small ribosomal subunit) is facing the viewer, with the 5S RNA at the top of the image. The large horizontal swath of “red” correlation co-localizes with the active site cleft that binds the mRNA, tRNAs, and translation factors. The first principal component (not shown) indicates a relatively simple, large-scale hinge-like motion in which the top third of the 70S subunit (in the orientation shown) is positively correlated with the bottom third.

### Structural Correlations during Evolution: Telomere End-Binding Proteins

Our method can also be used to analyze the correlated conformational changes that have occurred during the evolution of protein homologs. The ML superposition and first principal component for a set of homologous telomere end-binding protein OB-fold domains are shown in Figure 4. The PCA plot indicates a clear correlation between the two upper loops in blue and also within the red β-barrel, a fact that is otherwise difficult to ascertain from inspection of the structural alignment alone. The two blue loops are known to be critical for recognition of the proteins' single-stranded DNA ligand [17,18]. Thus, this PCA analysis implies that these loops (and also the β-barrel) have co-evolved in terms of conformation during the divergence of these domains from a common ancestor [19–21].

**Figure 4:**
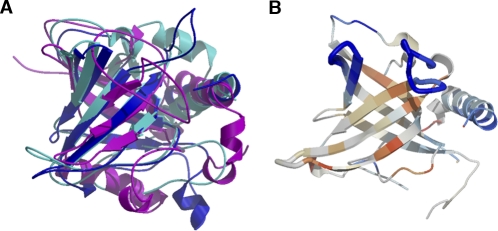
ML Superposition and PCA Plot of Homologous Telomere Domains (A) An ML superposition of the first OB-fold from 1otc (blue), 1s40 (magenta), and 1qzg (cyan). (B) A PCA plot of the first principal component based on the ML superposition in (A), plotted on the mean structure. Two functionally critical loops, shown in blue, are implicated in telomeric ssDNA substrate recognition. These loops are highly correlated, indicating that their conformations have evolved in concert.

### Structural Correlations within NMR Ensembles: DER of Ubiquitin

The correlations found in PCA plots are also useful for analyzing ensembles of solution structures of macromolecules solved by NMR spectroscopy. For instance, Figure 5A and 5C shows the largest principal mode of correlation from solution structures of ubiquitin solved by dynamic ensemble refinement, which takes into account the dynamic heterogeneity of a protein as measured by NMR relaxation experiments in addition to NOE distance constraint data [22]. Two independent NMR refinements of the ubiquitin structure are shown to give a sense of the reproducibility of our ML PCA method [22,23]. Two key residues in the core of the protein, Val5 and Ile30, pack against each other and are highly anti-correlated, indicating that during the “fluid-like” dynamic motion of the protein's interior these residues move in opposition to each other. Val5 and Ile30 are both members of a small set of core residues that have been implicated in forming a folding nucleus in ubiquitin [24]. Furthermore, these residues are notable for being some of the most highly conserved among ubiquitin homologs [25], for exhibiting the slowest rates of hydrogen exchange in the protein [26], and for decreasing the thermodynamic stability of the protein when mutated [27]. Together with these experimental results, ML PCA suggests that strongly correlated residues in ubiquitin are important for its folding and stability.

**Figure 5:**
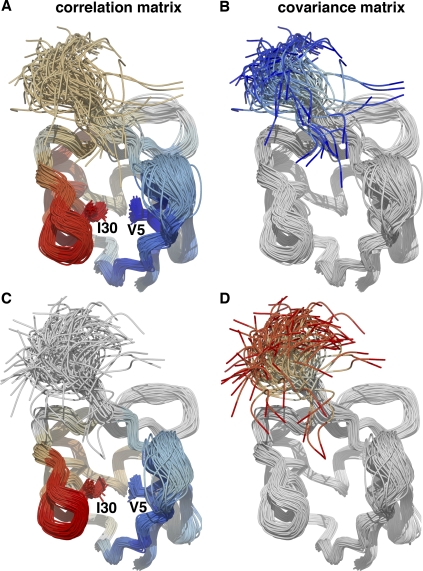
ML Superpositions and PCA Plots Derived from Two NMR Structural Ensembles of Ubiquitin (A) The first principal component from the *correlation* matrix is plotted on an ML superposition of the dynamic ensemble refined NMR solution structure of ubiquitin (PDB ID: 1xqq) [22], Ile30 (in red), and Val5 (in blue) pack together in the hydrophobic core of the protein. In this major mode of correlation, these two residues account for a large fraction of the correlation, and they move, on average, in opposite directions. (B) The first principal component from the *covariance* matrix, for the same 1xqq ensemble as in (A). The C-terminal tail (at top and in blue) is disordered largely because of a lack of NOE distance constraints. The low experimental precision of this region contributes to the large variance that dominates the largest principal component of the covariance matrix. The strong covariation in this region (indicated by blue) is thus an artifactual result of experimental uncertainty and dynamics rather than true correlated motion. (C) The first principal component from the *correlation* matrix is plotted on an ML superposition of an independent NMR solution structure of ubiquitin (PDB ID: 2nr2) [23]. (D) The first principal component from the *covariance* matrix, for the same 2nr2 ensemble as in (C). Note that in the disordered C-terminal tail the red versus blue color is arbitrary.

### Advantages of PCA of the Correlation Matrix versus the Covariance Matrix

Our method is reminiscent of previous work that has used PCA of covariance matrices to extract major modes of functionally relevant motions from MD trajectories [2–8]. However, the interpretation of PCA of a covariance matrix is problematic, as that method results in modes of covariation that are a convolution of both the correlation and the variance of the atoms (see Equation 3 in Methods). In structural superpositions, two very different factors contribute to the conformational variance: (1) random experimental uncertainties and (2) dynamic motion or conformational heterogeneity. Because we use the correlation matrix, rather than the covariance matrix, our method cleanly separates pure correlations from the variance, and thus the resulting principal components can be interpreted as bona fide modes of correlation.

For instance, often the variances in a covariance matrix are composed of stochastic contributions that can be physically irrelevant or uninteresting. In NMR ensembles, the variance of each atom reflects not only the dynamics of that atom but also the number of experimental constraints for the position of that atom. Highly uncertain regions of a structure can therefore dominate the largest principal component from a covariance matrix, thereby artifactually inflating the importance of these imprecise regions. An example is shown in Figure 5B, where the disordered C-terminal tail of ubiquitin has a large variance largely due to experimental imprecision (from a paucity of NOE distance constraints), resulting in its unilateral contribution to the largest principal component of the covariance matrix. PCA of a correlation matrix, on the other hand, circumvents this problem by down-weighting uncertain regions in proportion to their variances (see Equation 2 and compare Figure 5A and 5C with Figure 5B and 5D).

Furthermore, in an MD trajectory, a highly mobile loop with little correlated movement with other parts of the structure can nevertheless dominate the first mode of covariation. As a result, the largest principal components from the covariance matrix will primarily represent large magnitude motions with little or no real correlated movement. Covariance matrix PCA is useful, then, for analyzing major modes of motion when coordinate precision is high. However, covariance PCA is generally uninformative about true conformational correlation.

In sum, correlation matrix PCA produces modes of pure correlation that are independent of the uncertainties in atomic positions, since the variance components have been normalized away (Equation 2). Our ML method thus provides correlations that are unlikely to be artifacts of experimental imprecision or of the magnitude of dynamic motions in localized regions of the structure.

### Conclusion

Our maximum likelihood method provides principal components that accurately describe the modes of coordinated motions and correlations found in an ensemble of structures. By using correlation matrices rather than covariance matrices, the modes of correlation that are found are largely free of artifacts that can result from experimental imprecision and the magnitude of dynamic motion. Taken together, various experimental results suggest that highly correlated residues from PCA plots are likely to be functionally significant. Thus, maximum likelihood PCA of structural superpositions, and the structural PCA plots that illustrate the results, should prove to be of wide utility in analyzing and comparing macromolecules in diverse fields of structural biology.

## Methods

### Covariance and correlation matrices.

A covariance matrix is a mathematical description of the variation and covariation among members of a dataset. In the case of macromolecular structures, the covariance matrix describes the positional variation and correlations among the atoms observed in properly superpositioned family of structures. For example, given a protein *K* amino acids in length, here we consider the *K* × *K* covariance matrix representing the covariation of each of the *K* α-carbons with each of the others. If the orientations of the structures are known with certainty, then the diagonal elements *σ_i,i_* of the covariance matrix **Σ** are simply the variances for each of the atoms. Each off-diagonal element *σ_i,j≠i_* is the covariance of the *i* ^th^ atom with the *j* ^th^ atom. The elements *σ_i,j_* of the covariance matrix **Σ** can be defined as:

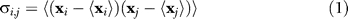
where 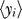 denotes the arithmetic average of *y_i_* over all *i*, and the **x** *_i_* here are 3-vectors representing the 3-D *x, y,* and *z* coordinates of each atom.

The correlation matrix **C**, on the other hand, is a simple function of the covariance matrix that has been normalized by the variances, leaving only pure correlations. Each element *c_i,j_* of the correlation matrix **C** is given by:

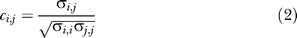
Unlike a covariance matrix, the diagonal elements of a correlation matrix all equal 1, and the non-diagonal elements range from −1 to 1 (corresponding to perfect negative correlation and positive correlation, respectively). Clearly, the accuracy of both the covariance and correlation matrices directly depends on the accuracy of the superposition. Note that, if the covariance matrix is known, then the correlation matrix is also necessarily known. However, the transform is not symmetric, as the correlation matrix does not contain all the information needed to reconstruct the covariance matrix; the variances are also required:

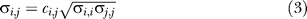



### Principal components analysis.

Major modes of structural correlation within a given structural dataset were found using the statistical method of principal components analysis (PCA). To perform PCA, the correlation (or covariance) matrix is diagonalized by spectral decomposition. The resulting eigenvectors are ranked according to their corresponding eigenvalues, largest to smallest. The eigenvector with the largest eigenvalue corresponds to the first principal component, which summarizes the major mode of correlation (or covariance) in the data. The second principal component corresponds to the second largest mode of correlation, and so on. Unless otherwise indicated, all examples reported here used PCA of the correlation matrix, although our program THESEUS will also perform PCA on the covariance matrix if desired (see Implementation).

### Theory.

*A statistical likelihood model for superpositioning structures.* A detailed treatment of the following likelihood analysis can be found elsewhere [12,13]. We present here a simplified account of the ML method and its rationale, focusing on simultaneous estimation of the covariance matrix in the macromolecular structural superpositioning problem. In the following, we specifically consider the superpositioning problem per se, as opposed to the structural alignment problem. We assume that the one-to-one correspondence between atoms or residues (i.e., the alignment) is known.

Consider superpositioning *N* different structures (**X** *_i_*, *i* = 1…*N*), each with *K* corresponding atoms. Each structure is mathematically represented as a *K* × 3 matrix of *K* rows of atoms. We assume a statistical perturbation model in which each macromolecular structure **X** *_i_* is drawn from a matrix normal (Gaussian) probability distribution [28,29]. Each structure **X** *_i_* to be superpositioned is considered as an arbitrarily rotated and translated Gaussian perturbation of a mean structure **M**:

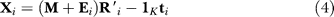
where ***t*** *_i_* is a 1 × 3 translational row vector, **1**_K_ denotes the *K* × 1 column vector of ones, and **R** *_i_* is an orthogonal 3 × 3 rotation matrix. The entries of the *K* × 3 matrix **E** *_i_* are filled with normal random errors, each with mean zero, i.e., **E** *_i_* ∝ N*_K_*_,3_(**0**, **Σ**, **I**_3_). The *K × K* covariance matrix **Σ** describes the (spherical) variance of each atom and the covariances among the atoms.

*The likelihood equation for matrix Gaussian superpositioning.* In the superposition problem with arbitrary translations, the covariance matrix **Σ** is poorly identified and singular unless it is parametrically constrained. Thus, to render the covariance matrix estimable, we assume that its eigenvalues are hierarchically distributed according to an inverse gamma probability density. An inverse gamma distribution is physically reasonable, as extremely small or large variances are relatively unlikely. The full joint log-likelihood for a structural superposition is then the sum of the log-likelihood for the eigenvalues of the atomic covariance matrix and the log-likelihood for the multivariate matrix normal density [30,31] corresponding to the statistical model given by Equation 4. The full superposition log-likelihood 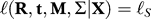 is thus

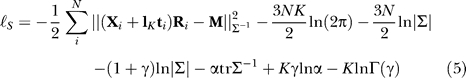
where |**U**| denotes the determinant of a matrix **U**, 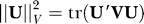 denotes a squared Frobenius Mahalanobis matrix norm, and *α* and *γ* are the scale and shape parameters, respectively, of an inverse gamma distribution for the *K* eigenvalues (*λ_j_*) of the atomic covariance matrix **Σ**:

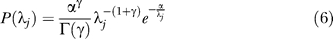



*ML superposition solutions.* In the following, we briefly give the ML solutions for each of the unknown parameters of the superposition log-likelihood equation from above.

Each observed structure must be translated to its row-weighted centroid:

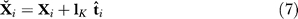
where **t̂" **_i_ is the ML estimate of the translation:

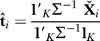



The optimal rotations are calculated using a singular value decomposition (SVD). Let the SVD of an arbitrary matrix **D** be **UΛV**'. Then, the ML rotations **R̂" **_*i*_ are estimated by

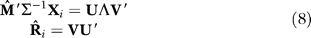



The mean structure is estimated as the arithmetic average of the optimally translated and rotated structures:

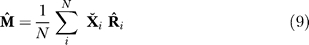



Finally, the ML estimate of the atomic covariance matrix 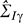 is given by:

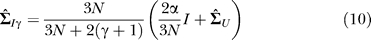
where the unconstrained ML estimate of the covariance matrix 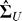 is:

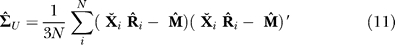



### Algorithm.

Because the estimate of the covariance matrix **Σ** is a function of the other unknown parameters, the ML solutions given above must be solved simultaneously by numerical methods [12,13]. We use an iterative algorithm based on the Expectation-Maximization (EM) method [32,33]. The algorithm assumes that the alignment (the one-to-one correspondence among atoms/residues in the structures) is known a priori, and it aims to determine the ML superposition given that alignment. In brief:

1. **Initialize:** Set 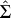 = **I**. Randomly choose one of the observed structures for use as the mean structure **M̂" **.

2. **Translate:** Translate (i.e., center) each according to Equation 7.

3. **Rotate:** Calculate each rotation **R̂" **_*i*_ (Equation 8), and rotate each translated structure by setting 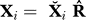 .

4. **Estimate the mean:** Recalculate the average structure **M̂" ** (Equation 9). Return to step 3 and loop until convergence.

5. **Estimate the inverse gamma distributed eigenvalues:** Estimate 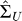 (Equation 11) and find its sample eigenvalues. Estimate the inverse gamma parameters by iteratively fitting them to the eigenvalues of the ML estimate of the covariance matrix, treating the zero eigenvalues (or the smallest variance) as missing data in an expectation-maximization algorithm.

6. **Estimate the atomic covariance matrix:** Modify 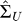 according to Equation 10. Return to step 2 and loop until convergence.

7. **PCA:** Perform a principal components analysis on the correlation matrix (or corresponding covariance matrix).

If all variances are assumed to be equal and all covariances are assumed to be zero (i.e., Σ ∝ I), then this algorithm corresponds to the classical least-squares algorithm for the simultaneous superpositioning of multiple structures [34–37]. The algorithm presented above (like that of Theobald and Wuttke [13]) is similar to that given in Theobald and Wuttke [12], with three exceptions. First, the algorithm of [12] is much more general, e.g., it is applicable to data in an arbitrary number of dimensions. Here we assume *D* = 3 for 3-D, spatial data. Second, here no scaling factors are necessary (i.e., *β_i_* = 1 for all structures), since molecules are inherently in the same scale, as bond lengths are fixed by the laws of physics. Third, we further assume that the variance about each atom is spherical (i.e., Ξ = I), an assumption that greatly simplifies the calculations.

### Implementation.

The algorithm described above for calculating ML superpositions and performing PCA of the estimated covariance matrix is implemented in the command-line UNIX program THESEUS [12,13]. THESEUS operates in two different modes: (1) a mode for superpositioning structures with identical sequences and (2) an “alignment mode,” which superpositions homologous structures with different residues given a known alignment (for instance, as determined from a sequence alignment program or from a structure-based alignment program). THESEUS does not perform structure-based sequence alignments, which is a distinct bioinformatic problem [38]. As with all superposition methods, THESEUS requires an a priori one-to-one mapping among the atoms/residues (i.e., it requires a known alignment). With NMR models or different crystal structures of identical proteins, the one-to-one mapping is trivial. When superpositioning different molecules with different sequences, however, a sequence alignment must be provided as a guide. THESEUS accepts sequence alignments in standard CLUSTAL and A2M (FASTA) formats.

In addition to the ML superposition for a set of structures, THESEUS will calculate the principal components of either the covariance or correlation matrix. For input, THESEUS takes a set of standard PDB formatted structure coordinate files (http://www.wwpdb.org/docs.html [39,40]). PCA analysis is requested with the “-P*n*” command line option, where “*n*” is substituted with the number of principal components desired (usually three are sufficient). PCA of the correlation matrix is performed by default; the “-C” option specifies that the covariance matrix should be used. Each principal component is written into the temperature factor field of two output files: (1) a PDB formatted file of the optimal ML superposition (each structure is represented as a different MODEL) and (2) a PDB formatted file of the estimate of the mean structure. Principal components can then be visualized as PCA plots (described in Results/Discussion) with any visualization software, such as PyMOL [41], RasMol [42], or MolScript [43], that can color the structures by values in the temperature factor field.

### Simulated structural data.

Two artificial datasets of protein coordinates were prepared as described previously [12]. Briefly, for each set, 300 protein structures were generated randomly, assuming a matrix Gaussian error distribution with a known mean protein structure and known atomic covariance matrix. The α-carbon atoms from model 1 of PDB:ID 2sdf (the human cytokine stromal cell-derived factor-1 protein [44]) were used as the mean protein structure (67 atoms/landmarks, squared radius of gyration = 152 Å^2^). The 67 × 67 atomic covariance matrices were based on values calculated from the superposition given in 2sdf, with variances ranging from 0.0452 to 79.2 Å and correlations from 0 to 0.99. Thus, in this simulation, the variances range over 3.2 orders of magnitude, a value that is typical for NMR solution structure ensembles (of 3,150 single-domain NMR families in the PDB database, the average range for the variance is 2.9 ± 1.1 (SD) orders of magnitude). The first simulated set of structures used a diagonal covariance matrix in which all covariances were set to zero. The second simulated set of structures used the full covariance matrix. Hence, both sets were generated with the same variances, differing only in their correlation structure. After generating the perturbed protein structures, each was then randomly translated and rotated.

Our ML superposition procedure was then performed on these simulated data sets, providing estimates of the atomic covariance matrix, along with estimates of the coordinates of the mean structure and of the original “true” superposition before translations and rotations had been applied. Default THESEUS parameters were used (version 1.2.6), except that the full covariance and correlation matrices were estimated with the “-c” command line option. For comparison, conventional least-squares superpositions were also calculated for the same dataset. The corresponding sample covariance and correlation matrices were calculated based on these least-squares superpositions. In order to show the effect of discarding a subset of highly variable (“disordered”) regions, separate least-squares analyses were performed using all atoms and also excluding residues 1–5 from the N-terminus, the atoms with the highest variance (referred to as “truncated least squares”).

### Illustrations.

Images of rendered macromolecules in Figures 2, 4, and 5 were made with POVScript+ [43,45] and Raster3D [46]. Figure 3 was made with PyMOL [41].
